# Supplementary material for: PAXIP1-AS1 is associated with immune infiltration and predicts poor prognosis in ovarian cancer
Source: PLoS One. 2023 Aug 15;18(8):e0290031. doi: 10.1371/journal.pone.0290031 (PMC10426951; doi:10.1371/journal.pone.0290031)
Supplement: Attachment — Submitted filename: Response to reviewers and editors.docx [file pone.0290031.s001.docx]

Dear Editor:

We have carefully studied the valuable comments from reviewers and the editor, and tried our best to revise the manuscript titled “PAXIP1-AS1 is associated with immune infiltration and predicts poor prognosis in ovarian cancer” (PONE-D-23-01712). The amendment has been marked with red. We have made detailed changes throughout the text. The point to point responds to the reviewer’s comments are listed in the revised text.

**Reviewer #1:**

In this paper, the authors comprehensively analyzed the effect of PAXIP1-AS1 expression on the prognosis of patients using OC gene expression and clinical information from the TCGA database, and verified the results by cell lines. In general, the author 's article box is clear, but the description and writing are confusing. The specific evaluation is as follows:

1. there are multiple OC datasets in the database, and it is recommended that the authors validate the results in other datasets.

**Response**: Thank you for your comments. GSE138866 was used to validate the prognostic value of PAXIP1-AS1 according to your suggestion.

2. The author 's method section is not clearly described, for example, it is simpler to describe in the data collection and processing stages.

**Response**: Thank you for your comments. Method section was improved according to your suggestion.

3. The format and typesetting of the whole article are very poor, and the size ratio of the picture is inconsistent.

**Response**: Thank you for your comments. The formatting and layout of the full text has been improved in accordance with the requirements of the journal.

4. In Figure 3, the tail of the survival curve clearly crossed, how the authors explained it.

**Response**: Thank you for your comments. The Logrank test does not require the proportional risk assumption to be met and can be used on an ad hoc basis when the proportional risk assumption is not met. The Logrank test was used in Figure 3 in this study. GSE138866 was used to validate the prognostic value of PAXIP1-AS1 (Figure 11) according to your suggestion.

5. In Figure 9, PAXIP1-AS1 did not correlate highly with a variety of immune cells, all below 0.3.

**Response**: Thank you for your comments. As you say, the absolute values of the correlation coefficients were all less than 0.3. This suggested a weak and positive or negative correlation between PAXIP1-AS1 and the infiltration of immune cells by both variants. However, a statistically significant association of PAXIP1-AS1 with the infiltration of immune cells could be determined on the basis of p-values. The description of the text was improved according to your suggestion.

6. It is well-known that the prognosis of tumors is more related to genomic variants, and whether the authors analyzed differences in genomic variants between patients in the high and low PAXIP1-AS1 expression groups.

**Response**: Thank you for your comments. The genomic variants between patients in the high and low PAXIP1-AS1 expression groups were supplemented according to your suggestion.

Thank you for considering our manuscript.

Yours sincerely,

Buze Chen
