# Supplementary material for: PAXIP1-AS1 is associated with immune infiltration and predicts poor prognosis in ovarian cancer
Source: PLoS One. 2023 Aug 15;18(8):e0290031. doi: 10.1371/journal.pone.0290031 (PMC10426951; doi:10.1371/journal.pone.0290031)
Supplement: Attachment — Submitted filename: Response to editors.docx [file pone.0290031.s002.docx]

Dear Editor:

We have carefully studied the valuable comments from the editor, and tried our best to revise the manuscript titled “PAXIP1-AS1 is associated with immune infiltration and predicts poor prognosis in ovarian cancer” (PONE-D-23-01712R1). The amendment has been marked with red. We have made detailed changes throughout the text. The point to point responds to the editor’s comments are listed in the revised text.

**Additional Editor Comments:**

Clinical information - No clinical info here, just data retrieval from TCGA; Data processing - No proper description, just data type conversion, how???; Differential expression of PAXIP1-AS1; The relationship between PAXIP1-AS1 and clinical characteristics; The relationship between PAXIP1-AS1 and prognosis; Gene set enrichment analysis (GSEA); Immune infiltration analysis by ssGSEA; Genomic variation between PAXIP1-AS1 high and low expression groups; Correlation between PAXIP1-AS1 and prognosis in GSE138866;----------- All the parameters, inputs, and output details need to be crystal clear.

**Response**: Thank you for your comments. The Materials and Methods section was improved according to your suggestion.

The specific steps were performed according to the reference [33, 34]----describe briefly.

**Response**: Thank you for your comments. The Materials and Methods section was improved according to your suggestion.

R software and statistical analysis - No proper description, just the name of the packages, meaningless. I suggest the author need to enclose all the scripts as an appendix or deposit them in GitHub if no copyright issues.

**Response**: Thank you for your comments. The Materials and Methods section was improved and the details of code and other original data were deposited in GitHub (https://github.com/BuzeChen15262020735/PAXIP1-AS1.git) according to your suggestion.

Thank you for considering our manuscript.

Yours sincerely,

Buze Chen
